# Real-Time Eye-to-Eye Contact Is Associated With Cross-Brain Neural Coupling in Angular Gyrus

**DOI:** 10.3389/fnhum.2020.00019

**Published:** 2020-02-06

**Authors:** J. Adam Noah, Xian Zhang, Swethasri Dravida, Yumie Ono, Adam Naples, James C. McPartland, Joy Hirsch

**Affiliations:** ^1^Brain Function Laboratory, Department of Psychiatry, Yale School of Medicine, New Haven, CT, United States; ^2^Interdepartmental Neuroscience Program, Yale School of Medicine, New Haven, CT, United States; ^3^Department of Electronics and Bioinformatics, School of Science and Technology, Meiji University, Kawasaki, Japan; ^4^Yale Child Study Center, Yale School of Medicine, New Haven, CT, United States; ^5^Department of Neuroscience, Yale School of Medicine, New Haven, CT, United States; ^6^Department of Comparative Medicine, Yale School of Medicine, New Haven, CT, United States; ^7^Department of Medical Physics and Biomedical Engineering, University College London, London, United Kingdom

**Keywords:** eye-to-eye contact, temporoparietal junction, two-person neuroscience, live dyadic interactions, fNIRS, hyperscanning, neural coupling, neural coherence

## Abstract

Direct eye contact between two individuals is a salient social behavior known to initiate and promote interpersonal interaction. However, the neural processes that underlie these live interactive behaviors and eye-to-eye contact are not well understood. The Dynamic Neural Coupling Hypothesis presents a general theoretical framework proposing that shared interactive behaviors are represented by cross-brain signal coherence. Using functional near-infrared spectroscopy (fNIRS) adapted for hyper scanning, we tested this hypothesis specifically for neural mechanisms associated with eye-to-eye gaze between human participants compared to similar direct eye-gaze at a dynamic video of a face and predicted that the coherence of neural signals between the two participants during reciprocal eye-to-eye contact would be greater than coherence observed during direct eye-gaze at a dynamic video for those signals originating in social and face processing systems. Consistent with this prediction cross-brain coherence was increased for signals within the angular gyrus (AG) during eye-to-eye contact relative to direct eye-gaze at a dynamic face video (*p* < 0.01). Further, activity in the right temporal-parietal junction (TPJ) was increased in the real eye-to-eye condition (*p* < 0.05, FDR corrected). Together, these findings advance a functional and mechanistic understanding of the AG and cross-brain neural coupling associated with real-time eye-to-eye contact.

## Introduction

Eye contact is a fundamental component of face-to-face communications and important in a number of developmental disorders including autism and psychiatric conditions (Pelphrey et al., 2005; Nation and Penny, 2008; Schneier et al., 2009; Senju and Johnson, 2009; McPartland et al., 2011; Jones and Klin, 2013). However, the neural mechanisms underlying direct eye-to-eye contact and its specific role in communication and social interaction are active areas of research. Technical developments in functional near-infrared spectroscopy (fNIRS) now enable broad acquisition of brain signals acquired simultaneously on two individuals under naturalistic conditions. Previous hyper scanning investigations of real (person-to-person) eye-to-eye contact compared with simultaneously viewing static face pictures using this technology have confirmed an association with language systems in the brain suggesting a link between eye contact and left hemisphere non-verbal communication systems (Hirsch et al., 2017). Other studies have shown roles for the inferior frontal gyrus, medial frontal gyrus, and occipito-temporal cortex involved in cross-brain interactions during up-regulation of attention and direct eye gaze (Lachat et al., 2012; Koike et al., 2016, 2019). In this study, we build on these advances to examine localized coherence responses of interacting dyads during real eye-to-eye contact, in contrast, to gaze at dynamic face videos. This is in contrast to previous work that focused on static photographs (Hirsch et al., 2017). We hypothesized that neural systems associated with socialization (Carter and Huettel, 2013) and dynamic face tracking (Pitcher et al., 2011a) would be associated with face and eye processing and that cross-brain coherence of neural responses would entrain face and social mechanisms between interacting pairs.

The perception of a dynamic face requires many complex factors to be interpreted in real-time to facilitate socialization and communication (Lachat et al., 2012; Koike et al., 2016, 2019; Chang and Tsao, 2017). Eye-to-eye contact is a dynamic and interactive behavior in which face cues are reciprocally exchanged and activity within neural networks specialized for facial recognition, dynamic motion, emotion, and socialization are expected to play a fundamental role. These networks include the temporal-parietal junction (TPJ), fusiform face area, occipital face area, and the posterior superior temporal sulcus (pSTS; George et al., 2001; Hooker et al., 2003; Mosconi et al., 2005; Pelphrey et al., 2005; Sorger et al., 2007; Saito et al., 2010; Cavallo et al., 2015). Additional anterior temporal gyrus and prefrontal lobe structures have also been shown to play a role in these interactions including the inferior and medial frontal gyri (Duchaine and Yovel, 2015). Neural activity specific to perception of faces has been observed in the inferior occipital and fusiform gyri, while perception of dynamic eye gaze has been associated with higher processing areas in the superior temporal sulci and TPJ (Haxby et al., 2000; Hoffman and Haxby, 2000; Pitcher et al., 2011a; Sato et al., 2016). While these areas have been shown to be involved in static and dynamic facial processing, the mechanism of information exchange and regulation of circuits that upregulate attentional mechanisms related to real and dynamic eye-to-eye contact between partners in social interaction is not well understood. Previous studies have explored the role of eye movement behaviors including blinking and attention regulation in a social circuit that is more active in joint attention tasks compared to simple eye gaze or during randomized video sequences (Lachat et al., 2012; Koike et al., 2016, 2019). Specifically, it was shown that neural synchrony across subjects was correlated with eye-blink synchronization (Koike et al., 2016) and that differences in alpha and mu oscillations in joint attention vs. no joint attention tasks suggested an increase in attention related to the social interaction (Lachat et al., 2012). The significance of these findings related to understanding the exchange of information in face-to-face interaction is enhanced by the relevance of eye contact behavior and social interaction difficulties that are characteristic of autism spectrum disorders (ASD), social anxiety, and schizophrenia (Schneier et al., 2009; Senju and Johnson, 2009; Tso et al., 2012).

The Dynamic Neural Coupling Hypothesis predicts that cross-brain coherence, calculated on residual, non-task related signals, represents a specific class of interactive functions characterized by exchange of rapid social information (Hasson and Frith, 2016). Evidence for coherence between neural circuits across partners has been observed during coordinated button pressing (Funane et al., 2011; Dikker et al., 2014); coordinated singing and humming (Osaka et al., 2014, 2015); gestural communication (Schippers et al., 2010); cooperative memory tasks (Dommer et al., 2012); and face-to-face unstructured dialogue (Jiang et al., 2012). Cross-brain coherence has also been previously shown to increase during live face-to-face interactions between dyads engaged in poker competitions in contrast to human-to-computer partners (Piva et al., 2017) in which cross-brain coherence specific to the human-to-human condition was observed between the angular gyrus (AG; a part of the TPJ) and occipito-temporal area, including the lateral aspect of the occipital and temporal lobes. This finding suggests a functional role for AG and face processing areas in coherent social interaction associated with face and eye processing and motivates the current investigation. It has been argued that increased neural synchrony or cross-brain coherence may represent changes in neural activity in the perceptual system of one brain which is coupled to the motor output system of another (Jacob, 2009; Dumas et al., 2010; Schippers et al., 2010; Koike et al., 2016).

Here, the specific neural responses across dyads while making eye contact were compared to when each subject alone interacted with a pre-recorded video of the face of a partner. In the case of the real partner, we hypothesize that detection of dynamic stimuli, such as facial expressions and eye movements known to occur in the real face condition, will elicit neural activity that is not present when subjects perform the same task with a pre-recorded video sequence of a dynamic face. Specifically, we predict increased cross-brain coherence of signals originating from areas of the cortex associated with visual and social functions.

## Materials and Methods

### Participants

Thirty healthy adults (15 pairs; 75% female; mean age 27.1 ± 8.5 years; 100% right-handed; Oldfield, 1971) participated in the study. All participants provided written informed consent in accordance with guidelines approved by the Yale University Human Investigation Committee (HIC #1501015178) and were reimbursed for participation. Dyads were not acquainted prior to the experiment and were assigned in order of recruitment.

### Stimuli and Procedures

**Figure 1:**
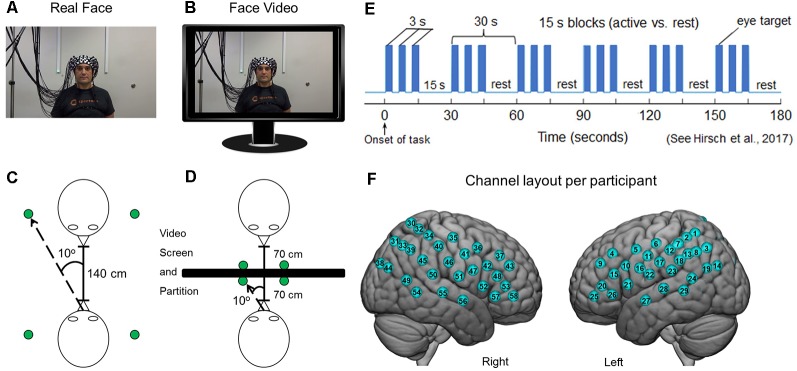
Experimental conditions. **(A)** Subjects were set up with 58 functional near-infrared spectroscopy (fNIRS) channels distributed bilaterally on the heads of both participants, who were seated across from each other so that each individual in a dyad could see the face of their partner. A small green Light Emitting Diode (LED) indicator lights located to either side of their partner indicated rest gaze targets. **(B)** Twenty-four-inch 16 × 9 monitors were placed between subjects and a size-calibrated, pre-recorded face video was presented in the same field of view as the live interaction. **(C)** Diagrammatic representation of dyadic interaction. Subjects were 140 cm apart from each other and the LED indicator lights placed 10° to the left and right of the face. **(D)** In the face-video condition, a partition was placed between subjects and monitors were arranged in the field of view of both partners. The face and LED sizes and positions were calibrated to subtend the same visual angles in both conditions. **(E)** Time course of the experimental paradigm. The entire duration of the run was 3 min and was repeated twice each for both the live interaction and the video face interaction. During the 3-min interaction, participants alternated between 15-s task and rest periods. In the task period, participants looked either directly at the eyes of their partner or at the left or right LED that was lit. During the rest period, subjects looked only at the lighted LED. The task was modified from one that has been used previously (Hirsch et al., 2017). **(F)** Optical channel layout for both hemispheres of each participant. The median locations of each channel are shown in Supplementary Table S1. Written informed consent was obtained from the individual for the publication of the images shown in panels **(A,B)**.

Each dyad participated in two tasks in which they were seated 140 cm across a table from each other. In both tasks, dyads alternated their gaze between the eyes of their partner and two small Light Emitting Diodes (LEDs) 10° to the left and to the right of their partner (Figure 1). In one condition, the partner was a real participant (Figure 1A), and in the other condition, the “partner” was a pre-rendered video of a person performing the same task (Figure 1B). In both conditions, dyad partners performed all tasks concurrently. The order of runs was randomly sequenced between viewing their real partner directly or viewing a visual-angle corrected video partner on a 24-inch 16 × 9 computer monitor placed back-to-back between subjects, including a partition to assure that dyads could not see their real partner during video conditions (Figures 1C,D). The face and distance of the video stimuli were calibrated to subtend identical degrees of visual angle in the field of view of the subjects and the timing and range of motion of eye movements between partners was the same in both tasks. A version of the time-series (Figure 1E) and experimental details are similar to a prior study (Hirsch et al., 2017). At the start of each task, an auditory cue prompted participants to gaze at the eyes of their real or recorded partner. Subsequent auditory tones alternatingly cued eye gaze between eyes or LED according to the protocol time series. The 15-s active task period alternated with a 15 s rest/baseline period. The task period consisted of three 6 s cycles in which gaze alternated on eyes for 3 s and on a lighted LED to either the right or left (alternating) of the subject for 3 s for each of three events. The time series was performed in the same way for all runs. The order of runs was counterbalanced across pairs of subjects. During the 15 s rest/baseline period, participants focused on the lighted LED, as in the case of the 3 s periods that separated the eye contact and gaze events. The 15 s activity epoch with alternating eye contact events was processed as a single block.

### Signal Acquisition and Channel Localization

Functional NIRS signal acquisition, optode localization, and signal processing, including global mean removal, were similar to methods described previously (Noah et al., 2015, 2017; Zhang et al., 2016, 2017; Piva et al., 2017; Dravida et al., 2018; Hirsch et al., 2018) and are summarized below. To assure that all participants provided recordable hemodynamic signals using fNIRS prior to participation in this experiment, subjects who demonstrated a significant fNIRS signal (*p* < 0.05) in the left motor cortex for both OxyHb and deOxyHb signals were eligible to participate in the present study. This technique assured that viable signals were recordable on all subjects.

Hemodynamic signals were acquired using three wavelengths of light, and an 80 fiber multichannel, continuous-wave fNIRS system (LABNIRS, Shimadzu Corporation, Kyoto, Japan). Each participant was fitted with an optode cap with predefined channel distances. Three sizes of caps were used based on the circumference of the heads of subjects. Large caps had a 60 cm circumference. Medium caps were 56.5 cm and small caps were 54.5 cm. Optode distances of 3 cm were designed for the 60 cm cap layout but were scaled equally to smaller caps. A lighted fiber-optic probe (Daiso, Hiroshima, Japan) was used to remove all hair from the optode channel prior to optode placement. Optodes consisting of 40 emitters and 40 detectors were arranged in a custom matrix, providing a total of 54 acquisition channels per subject. The specific layout with the coverage of the optode channels is shown in Figure 1F and the mean channel coordinates and locations are detailed in Supplementary Table S1. For consistency, placement of the most anterior channel of the optode holder cap was centered 1 cm above nasion. To assure acceptable signal-to-noise ratios, resistance was measured for each channel prior to recording, and adjustments were made for each channel until all recording optodes were calibrated and able to sense known quantities of light from each laser wavelength (Tachibana et al., 2011; Ono et al., 2014; Noah et al., 2015).

Anatomical locations of optodes in relation to standard head landmarks were determined for each participant using a Patriot 3D Digitizer (Polhemus, Colchester, VT, USA; Okamoto and Dan, 2005; Singh et al., 2005; Eggebrecht et al., 2012, 2014; Ferradal et al., 2014). Montreal Neurological Institute (MNI) coordinates (Mazziotta et al., 2001) for each channel were obtained using NIRS-SPM software (Ye et al., 2009), and the corresponding anatomical locations of each channel shown in Figure 1F was determined and detailed in Supplementary Table S1, which lists the group median MNI coordinates and anatomical regions with probability estimates for each of the channels.

### Signal Processing

Shimadzu LABNIRS systems utilize laser diodes at three wavelengths of light (780 nm, 805 nm, 830 nm). Raw optical density variations were translated into changes in relative chromophore concentrations using a modified Beer-Lambert equation (Hazeki and Tamura, 1988; Matcher et al., 1995; Hoshi, 2003). Signals were recorded at 30 Hz. Baseline drift was removed using wavelet detrending provided in NIRS-SPM (Ye et al., 2009). Global components attributable to blood pressure and other systemic effects (Tachtsidis and Scholkmann, 2016) were removed using a principal component analysis (PCA) spatial, global-mean filter (Zhang et al., 2016, 2017) prior to general linear model (GLM) analysis. Comparisons between conditions were based on GLM procedures using the NIRS-SPM software package. Event epochs within the time series (Figure 1E) were convolved with the hemodynamic response function provided from SPM8 (Penny et al., 2011) and were fit to the data, providing individual “beta values” for each participant across conditions. Group results based on these “beta values” were rendered on a standard MNI brain template (Figure 3). All analyses were performed on both Oxy- and deOxyHb signals (see Figure 3).

**Figure 2:**
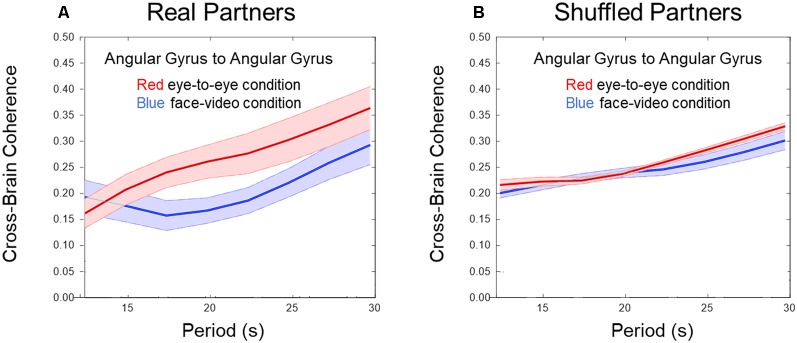
Cross-brain coherence. **(A)** Angular gyrus (AG) cross-brain coherence between paired dyads during direct eye-to-eye contact (red trace) shows increased coherence in the combined DeOxy+OxyHb signals for periods between 15 and 25 s compared to the face video condition (blue trace; *p* 0.01, *N* = 15 pairs). **(B)** Shuffled dyads do not show differences in cross-brain synchrony in the AG when comparing face-to-face or video face interactions.

**Figure 3:**
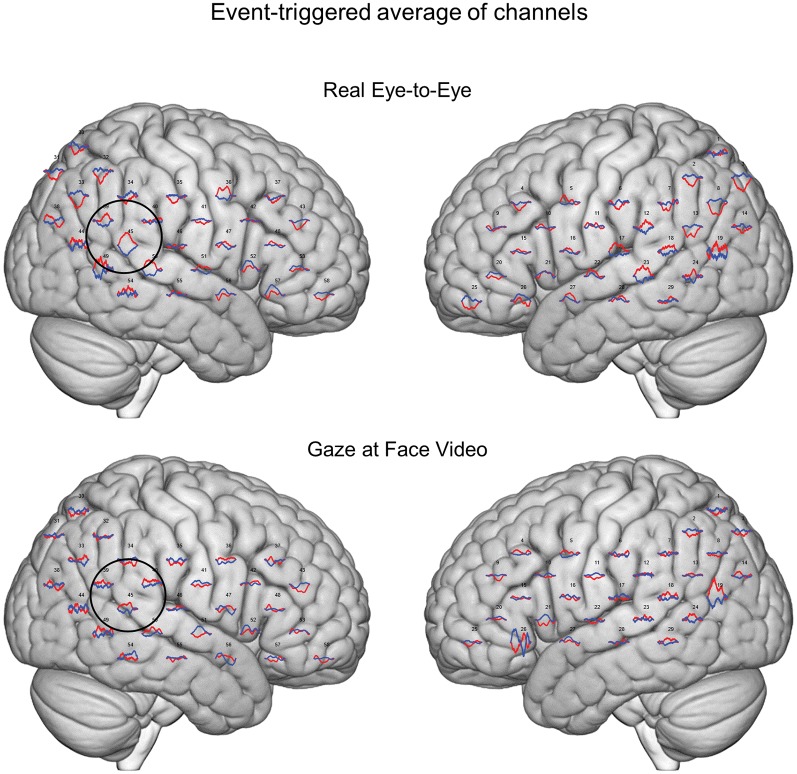
Event-triggered responses. Brain renders indicate event-triggered averaged eye-to-eye responses (top) compared to gaze at face video responses (bottom). Red traces represent group averaged OxyHb responses and blue traces represent deOxyHb responses. The circle represents a diagrammatic representation of the TPJ region of interest for the OxyHb signal (top) and deOxyHb (bottom) signal.

### Region of Interest: Temporal-Parietal Junction (TPJ)

Real-face and face-video conditions were compared using TPJ as a region of interest. The mask for the region was determined using Neurosynth (Yarkoni et al., 2011) and created through a meta-search performed for the term “TPJ.” Ninety-two results were found containing a total of 3,460 clusters. The mask was thresholded using a z-score of 6.3, and conditions were compared within this mask in the right hemisphere. To evaluate activity in the ROI determined in Neurosynth, each participant’s channel locations were first converted into MNI space (Dravida et al., 2018). Once in normalized space, a median beta value was determined within the mask and within a 1.8 cm depth from the cortical surface to use for subsequent analysis (Hirsch et al., 2018).

### Cross-Brain Coherence: Network of Interest

Cross-brain synchrony (coherence) was evaluated using wavelet analysis (Torrence and Compo, 1998; Cui et al., 2012) in the MATLAB 2018A Wavelet Toolbox. The wavelet kernel was a complex Gaussian provided by MATLAB. The number of octaves was four, and the range of frequencies was 0.4–0.025 Hz. The number of voices per octave was also four, and, therefore, 16 scales were used for which the wavelength difference was 2.5 s. Methodological details and validation of this technique have been previously described (Hirsch et al., 2017, 2018). Cross-brain coherence between dyads was measured between homologous pairs of brain regions using the combined Oxy- and deOxyHb signals. Individual channels were grouped into anatomical regions based on shared anatomy, which served to optimize signal-to-noise ratios. Grouping was achieved by identification of 14 bilateral ROIs from the acquired channels including: (1) AG (BA 39); (2) dorsolateral prefrontal cortex (BA 9); (3) dorsolateral prefrontal cortex (BA 46); (4) pars triangularis, BA 45; (5) supramarginal gyrus (SMG; BA 40); (6) middle temporal gyrus (MTG; BA 21); (7) superior temporal gyrus (STG; BA 22); (8) somatosensory cortex (BA 1, 2, and 3); (9) somatosensory association cortex (BA 7); (10) pre-motor and supplementary motor cortex (BA 6); (11) subcentral area (BA 43); (12) inferior frontal gyrus (BA 47); (13) visual cortex (Area V3, BA 19); and (14) frontal eye fields (BA 8). Signals acquired from predefined anatomical regions were decomposed into a range of temporal frequencies that were correlated across two brains for each dyad. This technique effectively removes the task regressor as is conventional for Psychophysiological Interaction (PPI) analysis (Friston et al., 1997). Here, we apply the decomposed “residual signal” to investigate effects other than the main task-induced effect. For example, cross-brain coherence of multiple signal components (wavelets) is thought to provide an indication of dynamic coupling processes rather than task-specific processes, which are coupled by virtue of the coordinated task. Coherence during eye-gaze was compared for face-to-face gaze and video-face gaze conditions. This analysis was also applied to shuffled dyads (random pairs). If the effects were due to social exchanges of salient cues, then the effects would be expected to disappear when partners were mixed (shuffled).

## Results

Figures 2A,B show cross-brain coherence (y-axis) and wavelet period in seconds (x-axis) for real and shuffled partners respectively comparing the eye-to-eye and face-video conditions. Red traces and shading indicate the mean ± SD in the live partner eye-to-eye condition, and blue traces indicate the face-video condition. An increase in coherence across live partners making direct eye-to-eye contact was observed in the AG between partners for temporal periods (wavelengths) between 15 and 25 s (*p* < 0.01). Figure 2B shows no difference in coherence between conditions when partners are shuffled, i.e., computationally paired with “partners” other than the real partner with whom he/she performed the task concurrently. Wavelet coherence was calculated for homologous regions. To further confirm the coherence results, we performed a permutation test between the 15 pairs of subjects and the two conditions. For this permutation test, we flipped the condition (face-to-face and video) for half the subjects and performed a *t*-test between the new mixed “conditions”. This procedure was repeated 1,000 times. The results of this permutation test showed 3.7% of trials produced type 1 error (rejection of the null hypothesis when it is really true) with similar significance as our result.

Averaged event-triggered responses for Oxy- and deOxyHb signals for the two conditions are shown in Figure 3. The top row shows the average localized responses for the real face-to-face task and the bottom row shows responses for the video gaze task. The black circle on the right hemisphere diagrammatically represents the TPJ. The hemodynamic responses with relative increases in OxyHb and decreases in deOxyHb can be seen in this region for the eye-to-eye condition compared to the video condition.

**Figure 4:**
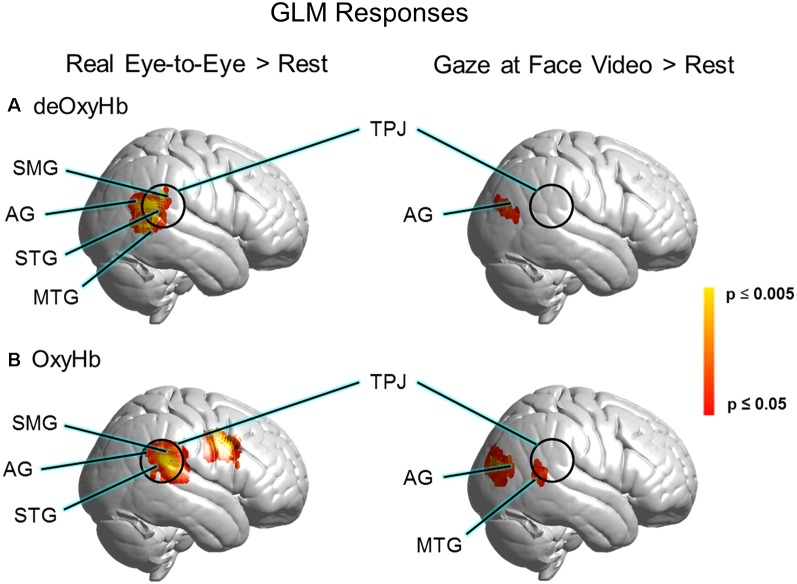
GLM responses for **(A)** deOxyHb and **(B)** OxyHb signals. The left column shows results for real eye-to-eye > rest. The right column shows results for gaze at face video > rest, respectively. GLM results for real eye-to-eye > rest (left column) show a cluster of activity located centrally in the right-lateralized TPJ region of interest, including the SMG, AG, middle temporal gyrus (MTG), and the STG (*p* 0.05, FDR-corrected, *N* = 30). Face video > rest fails to provide evidence for activity in the TPJ.

GLM comparisons are shown on brain renderings in Figure 4 for both the deOxyHb (Figure 4A) and the OxyHb (Figure 4B) signals (*N* = 30). Functional activity on the right hemisphere cortical surface for the eye-to-eye (left) and face-video (right) conditions are shown vs. rest (*p* ≤ 0.05, FDR-corrected). Findings are similar for both Oxy- and deOxyHb signals, and the deOxyHb signal is described in detail below because the deOxyHb signal is considered most similar to the blood oxygen level-dependent (BOLD) signal acquired by functional magnetic resonance imaging (fMRI; Strangman et al., 2002; Kirilina et al., 2012; Dravida et al., 2018). In the eye-to-eye vs. rest condition, a single cluster of activity was found including the STG, MTG, AG, and SMG with a peak MNI coordinate of 68, −46, 18, *T* = 4.67 and a *p*-value of 0.00003 (FDR-corrected, *p* ≤ 0.05). In the eye-to-video vs. rest condition, a single cluster of activity was found overlapping the right tertiary visual cortex and AG with a peak MNI coordinate of 48, −74, 18, *T* = 3.26 and a *p*-value of 0.0014.

**Figure 5:**
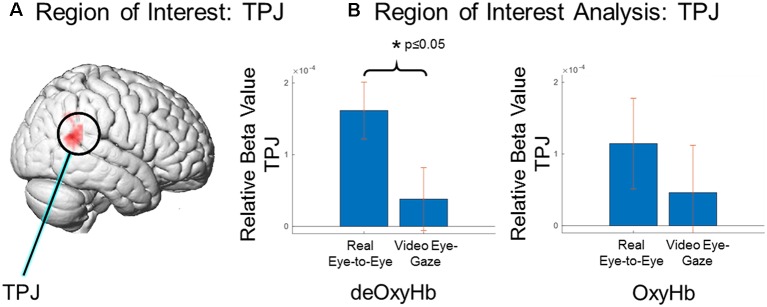
Region of interest comparison of signal strength (beta values) for eye-to-eye gaze at a real face and a pre-rendered dynamic face video. **(A)** Region of interest within the right TPJ as determined by Neurosynth (neurosynth.org). **(B)** Region of interest analysis: TPJ, Comparison of deOxyHb (left) and OxyHb (right) responses. The left bar in each graph shows results for real eye-to-eye > rest and the right bar in each shows results for gaze at face video > rest. ROI comparisons demonstrate the increased sensitivity of the deOxyHb signal (left) compared to the spatially-filtered OxyHb signal (right). However, both signals provide consistent and congruent findings.

A region of interest analysis based on the right TPJ (Figure 5A) was used to compare average signal strength (beta values) for the two conditions and two signals (Figure 5B). The real-eye > rest signals were greater than the video-gaze > rest for the deOxyHb signals. Average beta values in the ROI (paired *t*-test) yields a T statistic of 3.237 ± 1.63e^−04^ (*p* ≤ 0.05; Figure 5B, deOxyHb, left panel). OxyHb signals show a similar trend.

## Discussion

Increased cross-brain coherence between signals in the AG in the real eye-to-eye condition suggests that interactive and reciprocal behaviors between partners during eye contact increase activity in neural circuits associated with AG, a component of the TPJ. These results were specific only for eye-to-eye interactions (compared to watching a face video) and only occurred between interacting dyads (results on shuffled pairs showed no coherence). Increased coherence only in the live face-to-face task provides support for the hypothesis that reciprocal eye-contact dynamics between partners influences or modulates social network activity. A similar mechanism has been proposed by Tanabe and colleagues, suggesting an integrative role of the right STS in gaze processing, which has also been shown to be altered when individuals with autism interact with typically-developing subjects (Saito et al., 2010; Tanabe et al., 2012).

Both GLM (Figure 4) and ROI (Figure 5) results of the present study support and extend previous findings regarding the role of the TPJ in social interaction by demonstrating increased TPJ responses specific to dynamic face and eye contact in a live interaction. Real-time face-to-face interaction in a direct eye-gaze task activates this area to a greater extent during eye-to-eye contact with a live partner compared to the same task in a dynamic video face interaction with a pre-recorded video partner. The increased activity in the TPJ for the live condition that is not observed in the video condition supports the theoretical framework proposed in the Interactive Brain Hypothesis (De Jaegher et al., 2016), which purports that live interaction between individuals engages neural functions not engaged during similar tasks performed alone, i.e., without interaction. The increased activity in the right TPJ during the real eye task is consistent with sensitivity to social interaction in that region and suggests that these neural circuits reflect ecologically valid social activity highlighting the importance of two-person paradigms (Schilbach et al., 2013).

These findings advance a framework for interpersonal interaction that is linked to reciprocally shared dynamic content. We suggest that eye contact mediates information transfer between dynamic face and social areas across the brains of interacting dyads. The right-lateralized TPJ has been referred to as the hub of human socialization (Carter and Huettel, 2013) and shares overlapping functional responses to stimuli associated with visual discrimination of human or biological motion. For example, lateral temporal regions of the brain have been shown to display specialized responses to the motion of humans and objects (Beauchamp et al., 2002). The pSTS specifically responds more to human motion than object motion, and lateral temporal regions respond to the movement of humans and objects more than ventral temporal areas, which respond to static human and object stimuli. Lateral regions of superior temporal sulcus display specific responses to dynamic or moving faces in addition to motion of the whole body (Avidan et al., 2005). More recently, it has been suggested that the pSTS processes specific information regarding the dynamic aspects of faces, including movements of eye, mouth and head (Pitcher et al., 2011a,b). These findings advance our understanding of information transfer across individuals in the case of dynamic eye contact with cross-brain networks related to social interactions.

There are limitations to the interpretation of the results of this study. While the ROI analysis in this study showed activities specific to eye-to-eye interaction in the TPJ, other masks in additional ROIs were not investigated including the inferior and medial frontal gyri. These areas may also play a role in social attention. It is also possible that the mindset of individuals was not identical in both eye-to-eye and face video conditions. Differences in mindsets when looking at a live face and a video of a face may have provided additional social information and contributed to the increased activity in the TPJ. The spatial resolution of fNIRS (approximately 3 cm) does not allow discrimination of small anatomical differences in functional activity between gyri and sulci in similar locations, such as the STG vs. the neighboring sulci. Even with this limitation the results of this study show activity and connectivity specific to the superficial cortex, including the pSTS and the TPJ, during live interaction. Due to the optical methods of fNIRS, signals may contain systemic effects that originate from cardiovascular rather than neural sources (Tachtsidis and Scholkmann, 2016). Recent techniques that employ spatial filtering and short channel separation to remove these artifacts have been developed (Gagnon et al., 2014; Goodwin et al., 2014; Zhang et al., 2016, 2017). Here, when the spatial filtering technique was employed (Zhang et al., 2016, 2017) we found that the deOxyHb signals in the ROI analysis showed a significant difference between groups, and the OxyHb signals revealed a similar trend. Although event-triggered average results indicated localized concordance of Oxy- and deOxyHb signals associated with neural processing, the additional variance in the OxyHb signal (seen in the error bars in Figure 5) may have contributed to the lack of a significant difference, although a consistent trend is observed between the two signals. fNIRS has a penetration limit into the superficial gray matter of the cortex of around 2 cm. While we have access to the superficial face and eye areas on the occipital face area and TPJ, this limitation does not allow us to record from deeper structures involved in face processing, such as the medial structures of the fusiform face area. All reported findings are restricted to these superficial regions. Activities and coherence are also limited to temporal resolutions associated with hemodynamic responses. Future experiments could include methodologies that employ electroencephalography (EEG) and double density fNIRS to further investigate the relation of hemodynamic and electrocortical signals.

In conclusion, the findings of this experiment show increased task-related activity in the right TPJ in pairs of subjects that view each other face-to-face in real-time compared to when they perform an identical task with a pre-recorded video of a dynamic face. Further, increased coherence of signals in the AG (part of the TPJ) of both partners in the face-to-face condition suggests a link between eye-contact behavior and neural mechanisms of social interaction.

## Data Availability Statement

The datasets analyzed for this study will be made available upon request at fmri.org.

## Ethics Statement

The studies involving human participants were reviewed and approved by Yale Human Research Protection Program, Yale University. The patients/participants provided their written informed consent to participate in this study.

## Author Contributions

JN, XZ, SD, and JH designed and performed the experiment, analyzed the data, and wrote the manuscript. AN and JM assisted with experimental design. YO assisted with planning, data acquisition, and analysis.

## Conflict of Interest

The authors declare that the research was conducted in the absence of any commercial or financial relationships that could be construed as a potential conflict of interest.
