# Supplementary material for: Real-Time Eye-to-Eye Contact Is Associated With Cross-Brain Neural Coupling in Angular Gyrus
Source: Front Hum Neurosci. 2020 Feb 6;14:19. doi: 10.3389/fnhum.2020.00019 (PMC7016046; doi:10.3389/fnhum.2020.00019)
Supplement: Supplementary file 1 [file Table_1.DOCX]

**Supplemental Material**

| **Table S1. Channels, median coordinates, anatomical regions, & atlas-based probabilities** | | | | | | |
| --- | --- | --- | --- | --- | --- | --- |
| Channel | MNI Coordinates^1^ | | | Anatomical Region | BA^2^ | Probability |
| number | X | Y | Z |  |  |  |
| 1 | -42 | -61 | 59 | Somatosensory Association Cortex | 7 | 0.52 |
|  |  |  |  | Supramarginal Gyrus | 40 | 0.48 |
| 2 | -52 | -52 | 56 | Supramarginal Gyrus | 40 | 1.00 |
| 3 | -48 | -73 | 43 | Angular Gyrus | 39 | 0.64 |
|  |  |  |  | Visual Association Cortex, Area V3 | 19 | 0.19 |
|  |  |  |  | Somatosensory Association Cortex | 7 | 0.12 |
|  |  |  |  | Supramarginal Gyrus | 40 | 0.05 |
| 4 | -51 | 24 | 38 | Dorsolateral Prefrontal Cortex | 9 | 0.71 |
|  |  |  |  | Frontal Eye Fields | 8 | 0.16 |
|  |  |  |  | Dorsolateral Prefrontal Cortex | 46 | 0.13 |
| 5 | -59 | -1 | 42 | Pre- and Supplementary Motor Cortex | 6 | 0.95 |
|  |  |  |  | Dorsolateral Prefrontal Cortex | 9 | 0.05 |
| 6 | -61 | -23 | 49 | Primary Somatosensory Cortex | 2 | 0.36 |
|  |  |  |  | Primary Somatosensory Cortex | 1 | 0.26 |
|  |  |  |  | Pre- and Supplementary Motor Cortex | 6 | 0.15 |
|  |  |  |  | Primary Somatosensory Cortex | 3 | 0.12 |
|  |  |  |  | Primary Motor Cortex | 4 | 0.07 |
|  |  |  |  | Supramarginal Gyrus | 40 | 0.04 |
| 7 | -59 | -43 | 50 | Supramarginal Gyrus | 40 | 1.00 |
| 8 | -55 | -63 | 42 | Supramarginal Gyrus | 40 | 0.54 |
|  |  |  |  | Angular Gyrus | 39 | 0.46 |
| 9 | -50 | 35 | 28 | Dorsolateral Prefrontal Cortex | 46 | 0.90 |
|  |  |  |  | Dorsolateral Prefrontal Cortex | 9 | 0.09 |
| 10 | -62 | 10 | 26 | Dorsolateral Prefrontal Cortex | 9 | 0.43 |
|  |  |  |  | Pre- and Supplementary Motor Cortex | 6 | 0.26 |
|  |  |  |  | Pars Opercularis | 44 | 0.16 |
|  |  |  |  | Pars Triangularis | 45 | 0.15 |
| 11 | -65 | -12 | 37 | Pre- and Supplementary Motor Cortex | 6 | 0.61 |
|  |  |  |  | Primary Somatosensory Cortex | 3 | 0.18 |
|  |  |  |  | Primary Somatosensory Cortex | 1 | 0.09 |
|  |  |  |  | Primary Motor Cortex | 4 | 0.09 |
|  |  |  |  | Primary Somatosensory Cortex | 2 | 0.02 |
| 12 | -65 | -34 | 43 | Supramarginal Gyrus | 40 | 0.77 |
|  |  |  |  | Primary Somatosensory Cortex | 2 | 0.15 |
|  |  |  |  | Primary Somatosensory Cortex | 1 | 0.07 |
| 13 | -61 | -54 | 41 | Supramarginal Gyrus | 40 | 0.95 |
|  |  |  |  | Angular Gyrus | 39 | 0.05 |
| 14 | -48 | -82 | 26 | Angular Gyrus | 39 | 0.57 |
|  |  |  |  | Visual Association Cortex, Area V3 | 19 | 0.43 |
| 15 | -59 | 22 | 17 | Pars Triangularis | 45 | 0.69 |
|  |  |  |  | Pars Opercularis | 44 | 0.25 |
|  |  |  |  | Dorsolateral Prefrontal Cortex | 46 | 0.05 |
|  |  |  |  | Dorsolateral Prefrontal Cortex | 9 | 0.02 |
| 16 | -66 | -3 | 22 | Pre- and Supplementary Motor Cortex | 6 | 0.59 |
|  |  |  |  | Subcentral Area | 43 | 0.23 |
|  |  |  |  | Primary Motor Cortex | 4 | 0.10 |
|  |  |  |  | Superior Temporal Gyrus | 22 | 0.04 |
|  |  |  |  | Dorsolateral Prefrontal Cortex | 9 | 0.02 |
|  |  |  |  | Pars Opercularis | 44 | 0.02 |
| 17 | -68 | -25 | 31 | Supramarginal Gyrus | 40 | 0.50 |
|  |  |  |  | Primary Somatosensory Cortex | 2 | 0.28 |
|  |  |  |  | Primary Somatosensory Cortex | 1 | 0.15 |
|  |  |  |  | Primary Somatosensory Cortex | 3 | 0.07 |
| 18 | -66 | -45 | 33 | Supramarginal Gyrus | 40 | 1.00 |
| 19 | -55 | -73 | 25 | Angular Gyrus | 39 | 0.86 |
|  |  |  |  | Visual Association Cortex, Area V3 | 19 | 0.14 |
| 20 | -56 | 33 | 7 | Pars Triangularis | 45 | 0.49 |
|  |  |  |  | Dorsolateral Prefrontal Cortex | 46 | 0.25 |
|  |  |  |  | Inferior Frontal Gyrus | 47 | 0.24 |
|  |  |  |  | Frontopolar Area | 10 | 0.02 |
| 21 | -63 | 5 | 3 | Superior Temporal Gyrus | 22 | 0.63 |
|  |  |  |  | Pre- and Supplementary Motor Cortex | 6 | 0.14 |
|  |  |  |  | Pars Opercularis | 44 | 0.14 |
|  |  |  |  | Middle Temporal Gyrus | 21 | 0.09 |
| 22 | -68 | -15 | 16 | Subcentral Area | 43 | 0.45 |
|  |  |  |  | Primary and Associative Auditory Cortex | 42 | 0.36 |
|  |  |  |  | Superior Temporal Gyrus | 22 | 0.07 |
|  |  |  |  | Supramarginal Gyrus | 40 | 0.07 |
|  |  |  |  | Primary Somatosensory Cortex | 1 | 0.03 |
| 23 | -69 | -36 | 20 | Superior Temporal Gyrus | 22 | 0.49 |
|  |  |  |  | Supramarginal Gyrus | 40 | 0.38 |
|  |  |  |  | Primary and Associative Auditory Cortex | 42 | 0.13 |
| 24 | -65 | -55 | 16 | Superior Temporal Gyrus | 22 | 0.67 |
|  |  |  |  | Middle Temporal Gyrus | 21 | 0.15 |
|  |  |  |  | Supramarginal Gyrus | 40 | 0.10 |
|  |  |  |  | Angular Gyrus | 39 | 0.08 |
| 25 | -53 | 42 | -2 | Inferior Frontal Gyrus | 47 | 0.67 |
|  |  |  |  | Frontopolar Area | 10 | 0.12 |
|  |  |  |  | Dorsolateral Prefrontal Cortex | 46 | 0.12 |
|  |  |  |  | Pars Triangularis | 45 | 0.09 |
| 26 | -57 | 15 | -9 | Temporopolar Area | 38 | 0.52 |
|  |  |  |  | Superior Temporal Gyrus | 22 | 0.22 |
|  |  |  |  | Inferior Frontal Gyrus | 47 | 0.17 |
|  |  |  |  | Middle Temporal Gyrus | 21 | 0.09 |
| 27 | -68 | -7 | -10 | Middle Temporal Gyrus | 21 | 1.00 |
| 28 | -71 | -26 | 1 | Middle Temporal Gyrus | 21 | 0.47 |
|  |  |  |  | Superior Temporal Gyrus | 22 | 0.31 |
|  |  |  |  | Primary and Associative Auditory Cortex | 42 | 0.21 |
| 29 | -69 | -46 | 5 | Superior Temporal Gyrus | 22 | 0.54 |
|  |  |  |  | Middle Temporal Gyrus | 21 | 0.46 |
| 30 | 46 | -65 | 54 | Somatosensory Association Cortex | 7 | 0.63 |
|  |  |  |  | Supramarginal Gyrus | 40 | 0.35 |
|  |  |  |  | Angular Gyrus | 39 | 0.02 |
| 31 | 48 | -77 | 34 | Angular Gyrus | 39 | 0.59 |
|  |  |  |  | Visual Association Cortex, Area V3 | 19 | 0.41 |
| 32 | 54 | -57 | 53 | Supramarginal Gyrus | 40 | 0.96 |
|  |  |  |  | Somatosensory Association Cortex | 7 | 0.04 |
| 33 | 56 | -68 | 34 | Angular Gyrus | 39 | 0.89 |
|  |  |  |  | Supramarginal Gyrus | 40 | 0.11 |
| 34 | 63 | -45 | 47 | Supramarginal Gyrus | 40 | 1.00 |
| 35 | 63 | -22 | 49 | Primary Somatosensory Cortex | 2 | 0.30 |
|  |  |  |  | Primary Somatosensory Cortex | 1 | 0.26 |
|  |  |  |  | Pre- and Supplementary Motor Cortex | 6 | 0.18 |
|  |  |  |  | Primary Somatosensory Cortex | 3 | 0.16 |
|  |  |  |  | Primary Motor Cortex | 4 | 0.09 |
|  |  |  |  | Supramarginal Gyrus | 40 | 0.02 |
| 36 | 61 | 2 | 42 | Pre- and Supplementary Motor Cortex | 6 | 0.83 |
|  |  |  |  | Dorsolateral Prefrontal Cortex | 9 | 0.12 |
|  |  |  |  | Frontal Eye Fields | 8 | 0.04 |
| 37 | 52 | 27 | 39 | Dorsolateral Prefrontal Cortex | 9 | 0.69 |
|  |  |  |  | Frontal Eye Fields | 8 | 0.17 |
|  |  |  |  | Dorsolateral Prefrontal Cortex | 46 | 0.14 |
| 38 | 47 | -85 | 14 | Visual Association Cortex, Area V3 | 19 | 0.86 |
|  |  |  |  | Visual Association Cortex, Area V2 | 18 | 0.10 |
|  |  |  |  | Angular Gyrus | 39 | 0.04 |
| 39 | 63 | -58 | 32 | Supramarginal Gyrus | 40 | 0.63 |
|  |  |  |  | Angular Gyrus | 39 | 0.37 |
| 40 | 68 | -33 | 41 | Supramarginal Gyrus | 40 | 0.78 |
|  |  |  |  | Primary Somatosensory Cortex | 2 | 0.13 |
|  |  |  |  | Primary Somatosensory Cortex | 1 | 0.10 |
| 41 | 67 | -8 | 36 | Pre- and Supplementary Motor Cortex | 6 | 0.80 |
|  |  |  |  | Primary Somatosensory Cortex | 3 | 0.11 |
|  |  |  |  | Primary Motor Cortex | 4 | 0.06 |
|  |  |  |  | Primary Somatosensory Cortex | 1 | 0.03 |
| 42 | 63 | 13 | 26 | Dorsolateral Prefrontal Cortex | 9 | 0.51 |
|  |  |  |  | Pars Triangularis | 45 | 0.21 |
|  |  |  |  | Pars Opercularis | 44 | 0.18 |
|  |  |  |  | Pre- and Supplementary Motor Cortex | 6 | 0.10 |
| 43 | 52 | 36 | 29 | Dorsolateral Prefrontal Cortex | 46 | 0.85 |
|  |  |  |  | Dorsolateral Prefrontal Cortex | 9 | 0.15 |
| 44 | 54 | -77 | 12 | Visual Association Cortex, Area V3 | 19 | 0.71 |
|  |  |  |  | Angular Gyrus | 39 | 0.26 |
| 45 | 68 | -46 | 25 | Supramarginal Gyrus | 40 | 0.70 |
|  |  |  |  | Superior Temporal Gyrus | 22 | 0.30 |
| 46 | 70 | -23 | 29 | Supramarginal Gyrus | 40 | 0.43 |
|  |  |  |  | Primary Somatosensory Cortex | 2 | 0.21 |
|  |  |  |  | Primary Somatosensory Cortex | 1 | 0.20 |
|  |  |  |  | Primary Somatosensory Cortex | 3 | 0.09 |
|  |  |  |  | Subcentral Area | 43 | 0.05 |
|  |  |  |  | Pre- and Supplementary Motor Cortex | 6 | 0.02 |
| 47 | 67 | 1 | 22 | Pre- and Supplementary Motor Cortex | 6 | 0.64 |
|  |  |  |  | Subcentral Area | 43 | 0.09 |
|  |  |  |  | Dorsolateral Prefrontal Cortex | 9 | 0.08 |
|  |  |  |  | Primary Motor Cortex | 4 | 0.07 |
|  |  |  |  | Pars Opercularis | 44 | 0.06 |
|  |  |  |  | Superior Temporal Gyrus | 22 | 0.03 |
|  |  |  |  | Pars Triangularis | 45 | 0.03 |
| 48 | 61 | 26 | 17 | Pars Triangularis | 45 | 0.66 |
|  |  |  |  | Dorsolateral Prefrontal Cortex | 46 | 0.23 |
|  |  |  |  | Pars Opercularis | 44 | 0.10 |
| 49 | 66 | -58 | 7 | Middle Temporal Gyrus | 21 | 0.52 |
|  |  |  |  | Superior Temporal Gyrus | 22 | 0.25 |
|  |  |  |  | Angular Gyrus | 39 | 0.08 |
| 50 | 72 | -35 | 15 | Superior Temporal Gyrus | 22 | 0.62 |
|  |  |  |  | Primary and Associative Auditory Cortex | 42 | 0.29 |
|  |  |  |  | Supramarginal Gyrus | 40 | 0.08 |
| 51 | 70 | -12 | 13 | Subcentral Area | 43 | 0.36 |
|  |  |  |  | Primary and Associative Auditory Cortex | 42 | 0.32 |
|  |  |  |  | Superior Temporal Gyrus | 22 | 0.26 |
|  |  |  |  | Supramarginal Gyrus | 40 | 0.04 |
|  |  |  |  | Primary Motor Cortex | 4 | 0.02 |
| 52 | 64 | 9 | 3 | Superior Temporal Gyrus | 22 | 0.59 |
|  |  |  |  | Pars Opercularis | 44 | 0.24 |
|  |  |  |  | Pre- and Supplementary Motor Cortex | 6 | 0.10 |
|  |  |  |  | Pars Triangularis | 45 | 0.04 |
|  |  |  |  | Temporopolar Area | 38 | 0.02 |
|  |  |  |  | Inferior Frontal Gyrus | 47 | 0.01 |
| 53 | 58 | 35 | 7 | Pars Triangularis | 45 | 0.41 |
|  |  |  |  | Dorsolateral Prefrontal Cortex | 46 | 0.33 |
|  |  |  |  | Inferior Frontal Gyrus | 47 | 0.24 |
|  |  |  |  | Frontopolar Area | 10 | 0.02 |
| 54 | 70 | -48 | -3 | Middle Temporal Gyrus | 21 | 0.64 |
|  |  |  |  | Superior Temporal Gyrus | 22 | 0.16 |
| 55 | 73 | -24 | -1 | Middle Temporal Gyrus | 21 | 0.47 |
|  |  |  |  | Superior Temporal Gyrus | 22 | 0.40 |
|  |  |  |  | Primary and Associative Auditory Cortex | 42 | 0.14 |
| 56 | 69 | -5 | -9 | Middle Temporal Gyrus | 21 | 0.97 |
|  |  |  |  | Superior Temporal Gyrus | 22 | 0.03 |
| 57 | 59 | 19 | -7 | Inferior Frontal Gyrus | 47 | 0.43 |
|  |  |  |  | Temporopolar Area | 38 | 0.39 |
|  |  |  |  | Superior Temporal Gyrus | 22 | 0.16 |
|  |  |  |  | Pars Triangularis | 45 | 0.02 |
| 58 | 54 | 44 | -2 | Inferior Frontal Gyrus | 47 | 0.64 |
|  |  |  |  | Dorsolateral Prefrontal Cortex | 46 | 0.16 |
|  |  |  |  | Frontopolar Area | 10 | 0.14 |
|  |  |  |  | Pars Triangularis | 45 | 0.06 |
| ^1^Coordinates are based on the MNI system and (-) indicates left hemisphere. ^2^BA = Brodmann Area | | | | | | |

**Supplemental Table S1. Channel locations and labels.** Channel numbers (as shown on Fig. 1F) are listed in the left column. Anatomical locations of the channels are determined for each participant by digitization based on standard 10-20 fiduciary markers. Group-averaged (n = 30) centroids of the channels are based on the Montreal Neurological Institute (MNI) coordinate system (Mazziotta et al., 2001) and listed as x, y, z coordinates for which [-] values indicate left hemisphere. Anatomical regions, including Brodmann's Areas (BA) with probabilities of inclusion in the channel region/cluster, are shown in the center and right columns (52, 69).
